# Drug Effect of Clofazimine on Persisters Explains an Unexpected Increase in Bacterial Load in Patients

**DOI:** 10.1128/AAC.01905-19

**Published:** 2020-04-21

**Authors:** Alan Faraj, Robin J. Svensson, Andreas H. Diacon, Ulrika S. H. Simonsson

**Affiliations:** a Department of Pharmaceutical Biosciences, Uppsala University, Uppsala, Sweden; b TASK Applied Science, Cape Town, South Africa; c Division of Physiology, Faculty of Medicine and Health Sciences, Stellenbosch University, Tygerberg, South Africa

**Keywords:** *Mycobacterium tuberculosis*, drug development, pharmacodynamics, pharmacokinetics

## Abstract

Antituberculosis (anti-TB) drug development is dependent on informative trials to secure the development of new antibiotics and combination regimens. Clofazimine (CLO) and pyrazinamide (PZA) are important components of recommended standard multidrug treatments of TB. Paradoxically, in a phase IIa trial aiming to define the early bactericidal activity (EBA) of CLO and PZA monotherapy over the first 14 days of treatment, no significant drug effect was demonstrated for the two drugs using traditional statistical analysis. Using a model-based analysis, we characterized the statistically significant exposure-response relationships for both drugs that could explain the original findings of an increase in the numbers of CFU with CLO treatment and no effect with PZA. Sensitive analyses are crucial for exploring drug effects in early clinical trials to make the right decisions for advancement to further development. We propose that this quantitative semimechanistic approach provides a rational framework for analyzing phase IIa EBA studies and can accelerate anti-TB drug development.

## Abstract (precis)

Antituberculosis (anti-TB) drug development is dependent on informative trials to secure the development of new antibiotics and combination regimens. Clofazimine (CLO) and pyrazinamide (PZA) are important components of recommended standard multidrug treatments of TB. Paradoxically, in a phase IIa trial aiming to define the early bactericidal activity (EBA) of CLO and PZA monotherapy over the first 14 days of treatment, no significant drug effect was demonstrated for the two drugs using traditional statistical analysis.

## INTRODUCTION

Tuberculosis (TB) is the main cause of death from an infectious disease (1), and new drugs are urgently needed not only to shorten treatment but also to manage the rising numbers of cases with drug-resistant TB (2). Accompanying the need for new drugs, older approved drugs are repurposed (3, 4) to be included in new regimens, based on preclinical and clinical information.

Clinical symptoms appear when active infection is established. Stationary-phase infection in pulmonary tuberculosis is characterized by stable CFU counts over time of mycobacteria grown from sputum collected from untreated patients (5, 6). Experiments using resuscitation-promoting factors have emphasized that the majority of bacteria in a clinical sputum sample are noncultivable, nonmultiplying bacteria (7), which are undetectable using the number of CFU as a biomarker. However, the number of CFU is often applied in anti-TB drug development and phase IIa studies in particular. A decline in the number of CFU in the first days of treatment is generally considered a desirable treatment response, and a lack thereof makes the treatment appear unlikely to be clinically useful (8).

The aim of phase IIa TB trials is to assess the early antimycobacterial activity and safety in patients and guide informed decisions about which drug or regimen to move forward to more comprehensive and costly phase IIb trial evaluations. Usually, a phase IIa trial is designed to quantify the change in the mycobacterial load during the first 7 to 14 days of treatment. It is performed by quantifying early bactericidal activity (EBA), defined as the daily fall in the log_10_ number of CFU per milliliter of sputum (9). Further, empirical model-based approaches are frequently used to measure the change in the number of CFU. These include mono-, bi-, or multiexponential regression models (10, 11) and simultaneously involve all data from one patient in the estimation of the change in the number of CFU. It is generally accepted that a biphasic decline is due to a drug effect on different subpopulations of bacteria. As the types of bacteria exhibit different susceptibilities, an initial rapid decrease in the number of CFU occurs due to the effect on the most susceptible subpopulations, followed by a slower decline, representing the killing of the less susceptible subpopulation.

**FIG 1:**
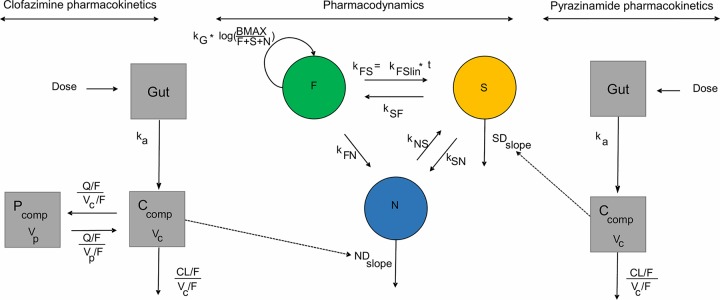
Schematic illustration of clofazimine and pyrazinamide pharmacokinetic models together with the multistate tuberculosis pharmacometric (MTP) model. *k_a_*, absorption rate constant; CL/*F*, apparent oral clearance; *V*/*F*, apparent volume of distribution; *C*_comp_, central compartment; *P*_comp_, peripheral compartment; *B*_max_, system carrying capacity; *k_FS_*, time-dependent linear rate parameter describing transfer from the multiplying (*F*) to the semidormant (*S*) state; *k*_SF_, transfer rate between the *S* and the *F* states; *k*_FN_, transfer rate between the *F* state and the persister (*N*) state; *k_SN_*, transfer rate between the *S* and the *N* states; *k_NS_*, transfer rate between the *N* and the *S* states; *k_G_*, growth rate constant. Dashed lines indicate the identified exposure-response relationship.

However, these models account only for the effects on viable bacteria that can grow CFU and not the drug effect on semidormant or nonmultiplying bacteria (persisters), which are thought to be the majority of the bacterial population and which do not grow on culture media used for assessing the number of CFU. The multistate tuberculosis pharmacometric (MTP) (12) model is a semimechanistic model combining three different bacterial subpopulations and the transfer between them (Fig. 1). Different bacterial subpopulations are defined by heterogeneity in metabolic activity, corresponding to fast multiplying (*F*), slowly multiplying (*S*), and nonmultiplying (*N*) bacteria, and for uniformity are referred to here as multiplying (*F*), semidormant (*S*), and persistent (*N*) bacteria. The MTP model has previously been successfully applied to preclinical (12, 13) and clinical (14) data for determination of exposure-response relationships, i.e., the drug effect. Initially developed on *in vitro* data, the MTP model approach has confirmed its role in translational medicine of TB, where exposure-response relationships based on *in vitro* information have successfully been used to predict the clinical trial response using clinical trial simulations (15, 16). Combined with the general pharmacodynamics interaction (GPDI) model (17, 18), a method for assessment of pharmacodynamic (PD) interactions, the MTP model has also successfully been applied to assessment of the PD interactions of anti-TB drugs both *in vitro* (19) and *in vivo* (16). The approach on which this work was built was selected by The Impact and Influence Initiative of the Quantitative Pharmacology (QP) Network of the American Society for Clinical Pharmacology and Therapeutics (ASCPT) and presented in a recent publication, which highlighted the most impactful examples of QP application, where QP played a transformational role, resulting in increased confidence in biomarker-driven decisions (20).

Clofazimine (CLO) and pyrazinamide (PZA) are established anti-TB drugs whose efficacy has been proven in clinical trials. CLO and PZA have also been studied in two monotherapy arms in a recent phase IIa trial (ClinicalTrials.gov registration number NCT01691534) (21). Although both drugs are recommended by the World Health Organization (WHO) as part of standard treatment regimens for drug-susceptible and drug-resistant TB (4), unexpectedly, no statistically significant drug effects were observed during 14 days of monotherapy. CLO, studied in an EBA study for the first time, even showed a numerical increase in the numbers of CFU (21). This was surprising, because CLO exhibits sterilizing activity in patients (22, 23) with multidrug-resistant TB (MDR-TB; defined as TB resistant to at least isoniazid [INH] and rifampin [RIF]). The paradoxical lack of EBA is well-known for PZA (6) and is in contrast to its ability to shorten TB treatment to 6 months when added to INH and RIF in the first 2 months.

In this work, the MTP model was linked to pharmacokinetic (PK) models and thereby used to investigate exposure-response relationships of the number of CFU after CLO and PZA monotherapy for 14 days to explain the paradoxical increase in the number of CFU during CLO treatment and to assess PZA monotherapy efficacy. The analysis revealed the significant activity of CLO and PZA against persistent and semidormant mycobacteria, respectively, that remained undetected with traditional methods of quantification of anti-TB drug effects.

## RESULTS

### Population pharmacokinetic modeling.

The final CLO PK model consisted of a two-compartment disposition model with first-order absorption and elimination. Additionally, a parameter explaining the lag time in absorption was supported by the data. No statistically significant covariate relationship was found using body weight, sex, or age on apparent oral clearance (CL/*F*) or apparent volume of distribution (*V*/*F*). Interindividual variability (IIV), expressed as the coefficient of variation (CV [in percent]), was supported for CL/*F* (75%), the first-order absorption rate constant (*k_a_*) (35%), and *V*/*F* (23%), whereas interoccasional variability (IOV) was statistically significant for bioavailability (*F*; 26%). The residual error model consisted of a proportional error model with a magnitude of 13.9%.

**FIG 2:**
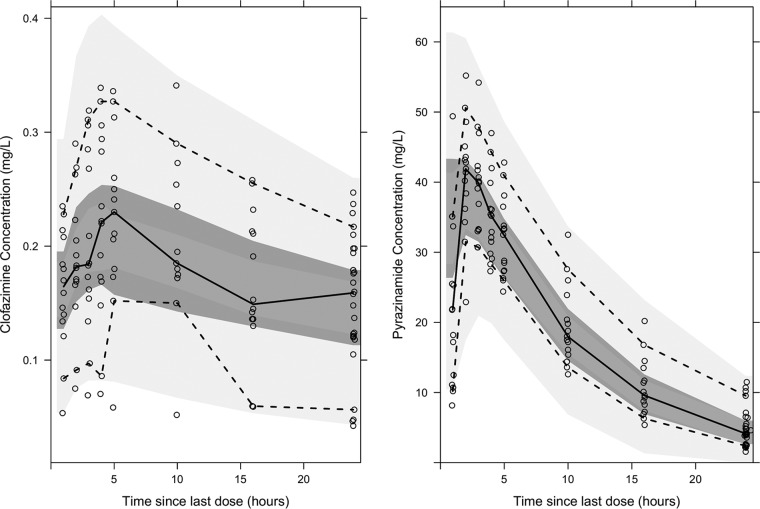
Visual predictive check for the observed CLO (left) and PZA (right) concentrations following rich sampling from day 14. Open circles are the observed data. The upper and the lower dashed lines illustrate the 90th and 10th percentiles of the observed data, respectively. The solid line is the median of the observed data. From top to bottom, shaded areas represent the 95% confidence intervals of the 90th percentile (light gray), the median (dark gray), and the 10th percentile (light gray) of the simulated data, based on 1,000 simulations.

A previously developed PK model for PZA (24) with a modest modification was found to describe the PZA PK data well. The original PK model included a bimodal distribution in *k_a_* values representing slow and fast absorbers, which was not supported by the data in this work. The data supported a unimodal distribution of *k_a_* values, corresponding to the fast-absorber proportion in the original publication. Hence, only the fast-absorber *k_a_* value was applied in this analysis. With this modest discrepancy, the earlier developed PK model (24) was able to predict population and individual PK profiles of PZA without reestimation, as seen in Fig. 2B and Fig. S1B in the supplemental material, respectively. All final PK parameter estimates are presented in Table 1.

**TABLE 1:** Parameter estimates based on the final models^*f*^

Parameter	Description	Estimate (% RSE)	% IIV (% RSE)	% IOV (% RSE)
Population PK parameters of CLO				
CL/*F* (liters·h^−1^)	Oral clearance	12.5 (145)	74.8 (160)^*a*^	
*V_c_*/*F* (liters)	Apparent volume of distribution	1,138 (18.4)	23.0 (85.9)^*b*^	
*k_a_* (h^−1^)	Absorption rate constant	0.67 (50)	35.3 (95.3)^*c*^	
*Q*/*F* (liters·h^−1^)	Intercompartmental clearance	63.3 (12.7)		
*V_p_*/*F* (liters)	Peripheral apparent volume of distribution	8,062 (82.7)		
*t*_lag_ (h)	Absorption lag time	0.62 (0.75)		
*F*	Bioavailability	1 (FIX)		43.8 (26.1)
Residual error parameter ε_prop_ (CV %)	Proportional error model parameter	13.9 (0.08)^*d*^		
MTP model parameters				
*k_G_* (days^−1^)	Fast multiplying bacterial growth rate	0.206 (FIX)		
*k_FN_* (days^−1^)	Rate of transfer from fast multiplyingto nonmultiplying state	8.98·10^−7^ (FIX)		
*k_SN_* (days^−1^)	Rate of transfer from slowly multiplying to nonmultiplying state	0.186 (FIX)		
*k_SF_* (days^−1^)	Transfer rate from slowly to fast multiplying state	0.0145 (FIX)		
*k_NS_* (days^−1^)	Transfer rate from nonmultiplying to fast multiplying state	0.00123 (FIX)		
*k_FS_*_lin_ (days^−2^)	Time-dependent transfer rate from fast to slowly multiplying state	0.00166 (FIX)		
*F*_0_ (ml^−1^)	Initial bacterial number of fast multiplying state	4.11 (FIX)		
*S*_0_ (ml^−1^)	Initial bacterial number in slowly multiplying state	9,770 (FIX)		
Exposure-response parameters of CLO				
ND_slope_ (liters·mg^−1^·days^−1^)	Second-order nonmultiplying state death rate	1.63 (11.5 [1.306–2.05]^*e*^)		
*B*_max_ (ml^−1^)	System carrying capacity per milliliter of sputum (COL arm)	0.06 × 10^9^ (35.1 [0.03 × 10^9^–0.10 × 10^9^])	133 (13.7 [93.1–157])	
Residual error parameters				
ε_add_ (% CV)	Additive residual error on log scale for all replicates	128 (7.85 [110–144])		
ε_repl_ (% CV)	Additive residual error on log scale between replicates	49 (17.4 [36.9–63.6])		
Exposure-response parameters of PZA				
SD_slope_ (liter·mg^−1^·days^−1^)	Second-order slow-multiplying state death rate	0.02 (30.6 [0.01–0.04])		
*B*_max_ (ml^−1^)	System carrying capacity per milliliter of sputum (PZA arm)	0.08 × 10^9^ (54.7 [0.04 × 10^9^–0.20 × 10^9^])	217 (16.3 [149–268])	
Residual error parameters				
ε_add_ (%) CV	Additive residual error on log scale for all replicates	98.5 (6.12 [87.7–108])		
ε_repl_ (%) CV	Additive residual error on log scale between replicates	39 (11.6 [31.4–46.4])		

a Shrinkage in IIV of apparent oral clearance, expressed in percent (10.5%).

b Shrinkage in IIV of the apparent volume of distribution, expressed in percent (16.5%).

c Shrinkage in IIV absorption parameter, expressed in percent (38.3%).

d Shrinkage in the proportional residual error model parameter, expressed in percent (15.6%).

e Values in brackets are the 90% confidence interval computed from the nonparametric bootstrap (*n* = 1,000).

f FIX, the parameter was fixed during estimation; RSE, relative standard error; IIV, interindividual variability, expressed as the coefficient of variation and as a percentage of the parameter estimate; IOV, interoccasional variability, expressed as the coefficient of variation and as a percentage of the parameter estimate. All MTP model parameters except *B*_max_ were fixed to estimates reported by Clewe et al. (12).

Visual predictive checks (VPCs) illustrating the observed data and how the final models adequately predicted the PK data were performed (Fig. 2A and B). As the pharmacokinetic-pharmacodynamic (PK-PD) effect evaluation was driven by input from the individual PK profiles for each patient, the observed and adequate model-predicted PK profile for each patient was important. The individual PK profiles of PZA and CLO were well predicted by the final PK models (Fig. S1).

### Pharmacokinetic-pharmacodynamic modeling.

The MTP model (12, 14) was used as the underlying disease model to describe the PD data (i.e., the numbers of CFU) from the two monotherapy treatment groups separately. All MTP model parameters except for the bacterial growth capacity of the system (*B*_max_) were fixed to estimates derived from *in vitro* natural growth data (12, 14). When modeling the CLO data, estimation of the baseline numbers of CFU, i.e., *B*_max_, resulted in a statistically significant drop in the objective function value (OFV; change in the objective function value [ΔOFV] = −3,212) compared to that obtained using the *in vitro* estimate, adjusting for the magnitude of the bacterial load in the clinical data on the numbers of CFU at stationary phase for the typical patient on CLO treatment. Introduction of IIV in *B*_max_ was statistically significant, as the OFV dropped by 106 points, enhancing the model fit and the functionality of the model, as it allowed for adjustment of the individual baseline numbers of CFU. Similarly, estimating *B*_max_ and implementation of IIV in *B*_max_ gave statistically significant OFV drops for the analysis of the numbers of CFU with PZA treatment.

The Bayesian *post hoc* PK estimates for each individual, based on the final PK model for each drug, were used as the input to the PK-PD modeling. A statistically significant exposure-response relationship (*P* < 0.05, OFV drop of 5.12) between adequately predicted individual CLO plasma concentrations, derived from the developed population PK model, and the killing of persistent bacteria was found. In this analysis, the discovered significant drug effect, denoted ND*_k_*, was a linearly concentration-dependent, second-order killing rate. The data did not support CLO killing of the other states alone or in combination. Further, the data did not support inhibition of the growth of the multiplying bacterial substate. The CLO drug effect after monotherapy identified in this analysis was in contrast to the findings of the analysis presented in the original publication (21), where no CLO drug effect in monotherapy was discovered when the number of CFU was used as a biomarker. This means that the original analysis could have missed significant drug effects of CLO on persisters, and if CLO had been a drug in development, it might have been unjustly abandoned.

**FIG 3:**
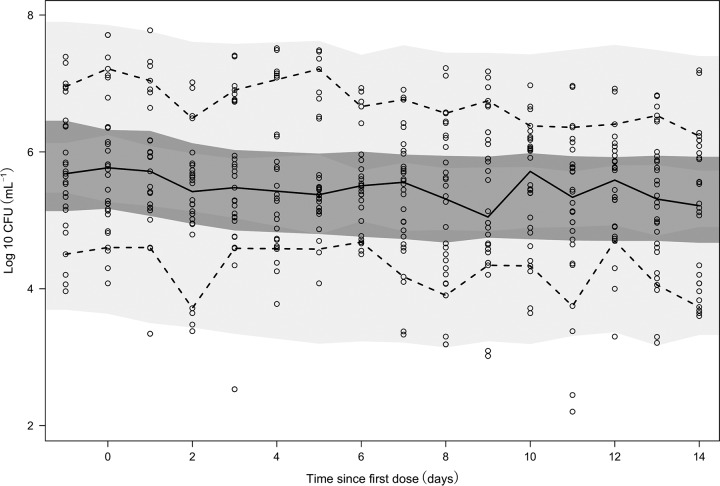
Visual predictive check of the final exposure-response model for patients receiving PZA. Dashed lines represent the 90th and 10th percentiles of the observed CFU data, whereas the solid line is the median of the observed CFU data. From top to bottom, the shaded areas represent the 95% confidence intervals of the 90th percentile (light gray), the median (dark gray), and the 10th percentile of the simulated data, based on 1,000 simulations. All open circles illustrate observation points.

For the exposure-response analysis of PZA in monotherapy, a statistically significant linear relationship (SD*_k_*) between individual PZA plasma concentrations and killing of the semidormant bacterial substate was found (*P* < 0.05, OFV drop of 5.81). This was in contrast to the analysis presented in the original publication (21), where no PZA drug effect in monotherapy was discovered using the number of CFU as a biomarker. The final exposure-response relationship predicted a decrease in the number of CFU over time (Fig. 3). The data did not support PZA inhibition of the bacterial growth or killing of the other states alone or in combination. This can explain the long-debated paradox that PZA exhibits a low EBA yet has proven to be able to shorten TB treatment to the current standard short course of 6 months.

The final differential equation system (equations 1 to 5) for the MTP model including the effects of CLO or PZA on the persistent or semidormant substate was as follows:

(1) $$\mathrm{dF}/\mathrm{dt}=k_{G}\cdot \text{log}\;\left[B_{\text{max}\;}/\left(F+S+N\right)\right]\cdot F+k_{\mathrm{SF}}\cdot S-k_{\mathrm{FS}}\cdot F-k_{\mathrm{FN}}\cdot F$$

(2) $$\mathrm{dS}/\mathrm{dt}=k_{\mathrm{FS}}\cdot F+k_{\mathrm{NS}}\cdot N-k_{\mathrm{SN}}\cdot S-k_{\mathrm{SF}}\cdot S-\text{ESD}\cdot S$$

(3) $$\mathrm{dN}/\mathrm{dt}=k_{\mathrm{SN}}\cdot S+k_{\mathrm{FN}}\cdot F-k_{\mathrm{NS}}\cdot N-\text{END}\cdot N$$

where *t* is time; *F*, *S*, and *N* are the numbers of bacteria (milliliter^−1^) in the *F*, *S*, and *N* states, respectively; *k_G_* is the growth rate constant; *k_SF_* is the transfer rate between the *S* and the *F* states; *k_FS_* is the time-dependent linear rate parameter describing transfer from the *F* to the *S* state; *k_FN_*, is the transfer rate between the *F* and the *N* state; *k_NS_* is the transfer rate between the *N* and the *S* states; and *k_SN_* is the transfer rate between the *S* and the *N* states and where

(4) $$\text{END}={\text{ND}}_{k}\cdot C_{\text{CLO}}$$

and

(5) $$\text{ESD}={\text{SD}}_{k}\cdot C_{\text{PZA}}$$

where END and ESD represent the linearly concentration-dependent drug effects for CLO and PZA, respectively; *C*_CLO_ is the CLO concentration; and *C*_PZA_ is the PZA concentration. The initial conditions of the differential equation system can be found in the NONMEM codes in Text Files S2 and S3. All final parameter estimates for the drug effects discovered using the MTP model as the underlying disease model can be found in Table 1.

**FIG 4:**
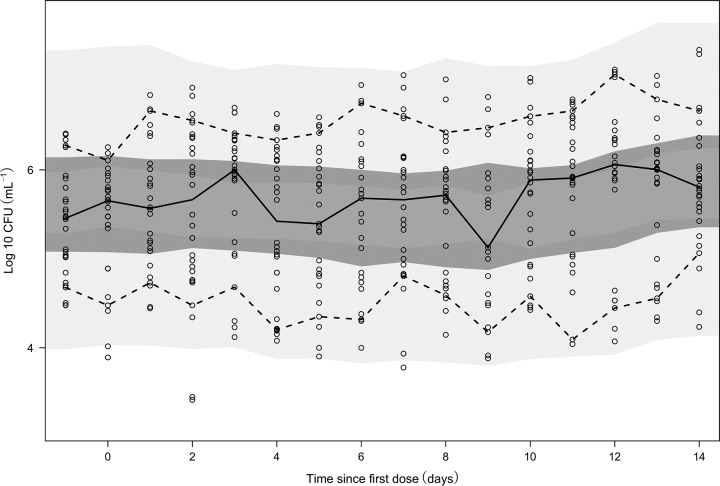
Visual predictive check of the final exposure-response model for patients receiving CLO. Dashed lines represent the 90th and 10th percentiles of the observed CFU data, whereas the solid line is the median of the observed CFU data. From top to bottom, the shaded areas represent the 95% confidence intervals of the 90th percentile (light gray), the median (dark gray), and the 10th percentile of the simulated data, based on 1,000 simulations. All open circles illustrate observation points.

The final MTP model provided adequate prediction of the observed numbers of CFU from patients in both treatment arms, as observed in Fig. 3 and 4, respectively. Typical bacterial simulations of each bacterial subtype and CFU counts can be found in Fig. S2.

### Sensitivity analysis.

The performed sensitivity analysis covered the impact of relative amounts between the different substates of bacteria at baseline on the final model. For instance, the *k_SN_* parameter was changed so that the persistent bacterial subtype consisted of 99% to 90% of total bacteria. No significant drop in the OFV (indicating that the alternative model did not improve the fit significantly) was observed following an empirical change of the original system parameters. This sensitivity analysis demonstrated that the CLO drug effect was statistically significant when the original system parameter estimates were used and that the model fit did not improve by empirically changing the system parameter estimates.

## DISCUSSION

In this work, the MTP model was utilized as a framework for studying antitubercular drug effects of CLO and PZA in monotherapy, using individual PK and PD (number of CFU) data from patients in a phase IIa study. In contrast to the primary analysis of the data (21), where no statistically significant rate of decline in the log_10_ CFU counts over the first 14 days of treatment were found, this analysis demonstrated statistically significant antimycobacterial activity for both CLO and PZA in monotherapy. The results indicate that the original analysis could have missed significant drug effects of CLO on persisters, and if the substance had been in development, it might have been wrongly rejected. Further, the results might explain the low EBA of PZA, even though it has been proven to shorten TB treatment to the current standard short treatment of 6 months. The results also indicate the increased statistical power obtained using the MTP model approach and emphasizes possible misinterpretations of potential drug effects when traditional statistical analysis is used. As the number of CFU is a summary biomarker only of multiplying bacilli, it is essential to emphasize that a drug that produces no decrease in the number of CFU in EBA studies using a one-population model may be a drug with efficacy against bacterial populations other than those able to grow CFU rather than one with no effect, as proposed in the original analysis. The findings are in accordance with previously published research (25), in which a simulated drug effect on the killing of the *N* substate (i.e., persisters) resulted in an indirect increase in the number of CFU. The results are also in line with those of earlier work showing that the MTP model approach gives a higher power to find statistically significant drug effects than traditional statistical analysis (25). The semimechanistic MTP model has previously been utilized to describe the drug effect on different TB bacterial substates *in vitro* (12), in mice (13), and with clinical data (14). Additionally, the MTP model has been externally validated for clinical trial simulations and proven to be able to predict a decrease in the number of CFU due to rifampin treatment (14).

Clofazimine was suggested to have a significant, linearly concentration-dependent effect on the persistent subpopulation. The data did not support any exposure-response relationship on the other bacterial substates, leaving the effect on the persistent state more interesting. Further, what makes this effect even more appealing is that it explains the numerical increase in the number of CFU seen in the original publication (21). A model-based explanation of the increase in the number of CFU lies in a regrowth phenomenon caused by the Gompertz growth function in the MTP model. The growth of the different bacterial subpopulations is constrained by a growth capacity, which is defined by the *B*_max_ term. When persistent bacteria are depleted, the density is decreased, paving the way for regrowth proportional to the decrease of the persistent state. Mathematically, the Gompertz function defines the growth of the multiplying state, according to equation 6:

(6) $$k_{G}\cdot \text{log}\;\left[B_{\text{max}\;}/\left(F+S+N\right)\right]$$

When persister (*N*-state) bacteria are killed, the growth of multiplying bacteria is enhanced due to a greater quota. This suggests an increase in the number of multiplying bacteria. As the number of CFU is a summary biomarker of multiplying (*F*)- and semidormant (*S*)-state bacteria, a total increase in the number of CFU is seen. However, it is important to recognize that this is the explanation directly inferred from the presented model. There may be alternative explanations that appear to be more mechanistically relevant. To our knowledge, this is the first clinically defined exposure-response relationship for CLO determined using EBA data.

In order to adjust for differences in the baseline number of CFU between the *in vitro* setting in which the MTP model was developed and the clinical data in this work, the parameter *B*_max_ was reestimated. Reestimation of *B*_max_ had no effect on the relative amounts of bacterial subpopulations but had an effect only on the baseline number of CFU. The relative amounts of multiplying, semidormant, and persistent bacterial substates were determined from the transfer rates between the subpopulations, which were fixed to *in vitro* estimates. The set of parameter values used resulted in a prediction of 99% persistent bacteria at baseline for a stationary-phase infection. This assumes that the relative amounts of the different subpopulations are the same *in vitro* and in patients. As a sensitivity analysis, the different transfer rates were empirically manipulated, resulting in different relative amounts, followed by reestimation of the CLO drug effect. No significant improvement was observed, which may provide further justification for the conclusion that CLO kills persistent bacteria (Fig. S2).

Clofazimine is a highly lipophilic antibiotic with a log partition coefficient (logP) value of >7 (26), exhibiting a long half-life. Previous reports suggest a half-life of 10 days after a single oral dose of 200 mg (27), while some reports suggest a half-life of >70 days with longer treatment (28). Due to the lipophilicity and high distribution into tissues, it is plausible and expectable that CLO exhibits a long half-life. Crystal-like inclusions composed of CLO have been reported in several tissues *in vivo* (29) and in patients (30), which demonstrates a capability to accumulate. As absorption into plasma is vastly dependent on the concentration of dissolved molecules in solution, the lipophilic nature of CLO causes a large variability in absorption due to poor solubility in physiological fluids (31).

IIV was supported by the data for CL/*F*, *V*/*F*, and *k_a_*, whereas IOV was included in bioavailability (*F*). Due to the physiochemical and PK properties of CLO discussed above, it is expected that bioavailability varies between individuals and occasions of intake. As oral clearance is dependent on bioavailability and could be affected by *k_a_*, a high variability in absorption might have been the reason for the high uncertainty in the estimated typical CL/*F* value. Reports have indicated significant food effects on the bioavailability of CLO, showing a 45% increase when CLO is administered with a high-fat meal compared to that when it is administered in a fasting state (31). Rich PK sampling on day 14 revealed high variability in exposure data (see Fig. S3 in the supplemental material). The highly variable nature of the PK properties of CLO introduced uncertainty into the estimated population PK parameters, as the total number of subjects was low. However, for the subsequent PD analysis using the MTP model, this was not a problem, as the adequately model-predicted individual PK profiles were used for the input into the evaluation of drug effects.

As a TB culture or infection enters stationary phase, the majority of the bacteria may not grow on solid media (32). Alternatively, they may grow better in liquid media. It has been demonstrated for a high-dose rifampin trial that one subpopulation of TB was quantifiable in liquid media but not on solid media (where rifampin exhibited a dose-dependent effect on this subpopulation) (33). The persistent (*N*) state of the MTP model may partly or completely correspond to the considered bacterial subpopulation that can grow in liquid culture. The effectiveness and low prevalence of resistant strains against CLO could be due to the fact that it was rarely used for the treatment of TB in the past, it has several molecular mechanisms of action (34, 35) on persistent bacteria, and it has a propensity to accumulate in tissue. As CLO exhibits high lipophilicity, it is expected to penetrate lesions and has been demonstrated to kill hypoxic nonreplicating bacteria *in vitro* (36). Furthermore, it has been demonstrated to exhibit membrane-destabilizing properties, an effect that was attenuated in the presence of membrane-stabilizing agents (37). In a clinical setting, the addition of CLO to multichemotherapy regimens resulted in significantly higher sputum culture conversion compared to individualized background treatment (22). Furthermore, the treatment success rate was higher in the CLO-containing regimen, with cavity closure occurring earlier than it did with individualized background treatment. However, the exact mechanism of CLO-mediated antimicrobial activity needs further investigation.

In contrast to the original analysis of the PZA CFU data, a statistically significant linearly concentration-dependent effect for the killing of semidormant bacteria following PZA treatment was discovered. The effect was evaluated using adequately predicted individual exposures. These findings are in accordance with those of previous research stating that infected macrophages contain phagolysosomes with a low pH, an environment that results in semidormant bacteria as well as the activation of PZA (38). As an acidic environment shifts bacterial metabolism and creates circumstances adequate for the PZA drug effect, the discovered findings are plausible. These findings are also in accordance with the clinical situation, in which PZA is effective during the first 2 months of the standard multidrug anti-TB treatment, potentially eradicating the majority of slow multipliers. Furthermore, PZA is metabolized in the tubercle bacterium into pyrazinoic acid, an active metabolite that has shown a capability to distribute into lesions to the same extent as PZA (38). As stated by the original authors, PZA exhibits a slight EBA when biomarkers other than the number of CFU are used, but this work demonstrates a statistically significant drug effect when the numbers of CFU are used as well.

In the investigation of exposure-response relationships, it is favorable to have PK and PD (number of CFU) data from the same individuals, which, fortunately, was the case for this clinical study. Primarily, adequate individual model-predicted PK profiles relative to the provided PK data were ensured and subsequently used to evaluate drug effects. Most phase IIa trials aiming to investigate EBA in TB research involve 10 to 15 patients per arm (9). Despite this small number of patients, the translational MTP model demonstrated a higher power than the traditional statistical analysis performed in the original study. This demonstrates a clear advantage when a semimechanistic PK-PD model, such as the MTP model, is used rather than when traditional statistical methods are used. The reason for the increased power is the use of a nonlinear mixed effects approach, in which all data are analyzed simultaneously, and the fact that the analysis includes a semimechanistic structure of not only multiplying substates but also a nonreplicating state, which makes up a majority of the total bacterial burden. Due to the parameters of the semimechanistic MTP model, it was possible to estimate a drug effect of CLO that suggests mycobacterial killing, even though the numbers of CFU increased. Using a traditional analysis, an increase would not be interpreted as the drug being effective, whereas the MTP model approach includes all data simultaneously, including individual PK exposures, and does not constrain the analysis to a decrease in the numbers of CFU. To avoid unperceived exposure-response relationships in the data, the model-based analysis utilized a PK-PD approach and previously demonstrated a higher statistical power than traditional statistical analysis (25, 39, 40). As the sizes of phase IIa trials in anti-TB drug development constrain the definition of such relationships using traditional analysis methods, it is important to emphasize more sensitive and modern methodologies. By reducing the number of patients required to detect significant drug effects, anti-TB drug development could be less costly and, therefore, enhanced. Although the model was developed on *in vitro* data, it is translationally capable of describing clinical data, as in the case of the CLO drug effect. However, in order to rationally implement these findings in the clinic, further clinical trials investigating the CLO drug effect are desired. To define CLO PK and its variability components with higher precision, additional data are needed.

In contrast to the original analysis, statistically significant drug effects were discovered for both PZA and CLO in monotherapy using the number of CFU as a biomarker. The drug effect on persistent tubercular bacilli explains the unexpected increase after CLO monotherapy and sheds light on possible misinterpretations of drug effects when the number of CFU is used as a biomarker together with traditional statistical analysis. Thus, the MTP model can be utilized for analysis and simulation of clinical trials to accelerate anti-TB drug development.

## MATERIALS AND METHODS

### Patients and study design.

Data were obtained from a 14-day phase IIa, two-center, open-label, randomized clinical trial including a PZA treatment arm (1,500 mg once daily [o.d.]; *n* = 15) and a CLO treatment arm (300 mg o.d. on days 1 to 3 and 100 mg o.d. on days 4 to 14; *n* = 14) (21). Counts of Mycobacterium tuberculosis CFU were quantified daily from sputum collected 16 h (overnight) from 2 days before the start of treatment to the last treatment day. PK plasma sampling was conducted hourly from predose to 5 h postdose on days 1, 2, 3, and 8, with rich sampling being performed on day 14 by also including collection time points of 10, 16, and 24 h after dosing. All patients were adults with confirmed treatment-naive pulmonary TB. Ethical clearance was obtained from the local ethics committee, and informed consent was obtained from all patients prior to the study. The trial was conducted in accordance with good clinical practice and was approved by the Research Ethics Committee, University of Cape Town, Cape Town, South Africa, and Pharma Ethics Pty. Ltd., South Africa. More detailed information regarding the clinical trial design and baseline characteristics can be found in the original publication (21).

### Population pharmacokinetic modeling.

One- and two-compartment disposition models were tested for CLO. Based on previously published work (31), a parameter accounting for an absorption lag time was explored. To account for the difference in dosage between days 1 to 3 and days 4 to 14, a relative bioavailability (*F*), expressed as a quota, was tested and compared to a scenario in which typical bioavailability was fixed to a value of 1. The stochastic model was developed by exploring IIV and IOV, alone and in combination, in all structural parameters. Nonsignificant parameters were not included. Proportional and/or additive residual error models were implemented, to elucidate the appropriate error model for the data. Allometric scaling was applied on oral clearance, intercompartmental clearance (*Q*/*F*), and both the apparent volume of distribution in plasma (*V_c_*/*F*) and the apparent volume of distribution in the periphery (*V_p_*/*F*), as described by Anderson and Holford (41).

(7) $${\text{CL}}_{i}={\text{CL}}_{\text{typ}}\cdot {\left({\text{WT}}_{i}/53\right)}^{0.75}$$

(8) $$V_{i}=V_{\text{typ}}\cdot {\left({\text{WT}}_{i}/53\right)}^{1}$$

where CL*_i_* and *V_i_* are scaled typical values of CL and *V* for individual *i*, respectively. CL_typ_ and *V*_typ_ correspond to CL and *V* parameters for a typical individual of 53 kg (median weight of the study population). WT*_i_* is the body weight of individual *i* (in kilograms). A notation similar to that observed in equations 7 and 8 was implemented for *Q* and *V_p_*. Further, body weight, age, and sex were tested as covariates on CL/*F* and *V*/*F*.

For the PZA PK data, a previously developed population PK model was utilized without the reestimation of population parameters (24). In brief, the PZA model consisted of a one-compartment distribution model with first-order absorption and elimination to and from the central compartment, respectively. The model included a component to account for a bimodal distribution of *k_a_* values and was therefore evaluated. Further, the model included a zero-order rate constant accounting for the release of drug from the formulation, expressed as the input into the absorption compartment. IIV was included in CL/*F*, *V*/*F*, and the duration of the zero-order release from the formulation. IOV was included in CL/*F* and *k_a_*. Residual variability was described using a combined-error model consisting of a proportional error at high concentrations and an additive error component at lower concentrations. Body weight was included as a covariate on CL/*F* and *V*/*F*, while sex was included as a covariate on *V*/*F*.

### Pharmacokinetic-pharmacodynamic modeling.

The MTP model (12), originally developed on *in vitro* data, was applied to the clinical CFU counts from the different treatment arms as a disease model to explore significant drug effects on the different bacterial subpopulations. The number of CFU was predicted based on the sum of the sizes of the multiplying (*F*) and semidormant (*S*) bacterial substates, whereas the persistent (*N*) bacterial substate was considered nonmultiplying on solid medium and as such was in a hidden state (42). In brief, the MTP model consists of a differential equation system accounting for the transfer rates and conversion from one substate to another and reflected a change in metabolic activity. The assumed scientifically plausible direction of flows can be observed in Fig. 1, as well as in the following differential equations (equations 9, 10, and 11), which defined the MTP system:

(9) $$\mathrm{dF}/\mathrm{dt}=k_{G}\cdot \text{log}\;\left[B_{\text{max}\;}/\left(F+S+N\right)\right]\cdot F+k_{\mathrm{SF}}\cdot S-k_{\mathrm{FS}}\cdot F-k_{\mathrm{FN}}\cdot F$$

(10) $$\mathrm{dS}/\mathrm{dt}=k_{\mathrm{FS}}\cdot F+k_{\mathrm{NS}}\cdot N-k_{\mathrm{SN}}\cdot S-k_{\mathrm{SF}}\cdot S$$

(11) $$\mathrm{dN}/\mathrm{dt}=k_{\mathrm{SN}}\cdot S+k_{\mathrm{FN}}\cdot F-k_{\mathrm{NS}}\cdot N$$

in which the rate constant *k* is labeled with two-letter subscripts, with the first letter representing the origin and the second letter representing the flow direction. The transition rate from multiplying (*F*) to semidormant (*S*) bacterial substate, denoted $k_{\mathrm{FS}}=k_{\mathrm{FS}\text{lin}}\cdot t$ (where *k_FS_*_lin_ is the time-dependent transfer rate from the fast to the slowly multiplying state) was unique, in terms of the time (*t*) after infection (days) dependency. Growth of the multiplying (*F*) state (*k_G_*) was accounted for using a Gompertz function. *F*, *S*, and *N* were the model-predicted bacterial number (milliliter^−1^) in the multiplying, semidormant, and persistent states, respectively. All transitions were allowed to occur, except for the flow of the persistent state to the multiplying state, although this is indirectly possible through the transition to a semidormant state. As represented in Fig. 1 and in equations 9, 10, and 11, direct growth of the multiplying (*F*) state was possible, whereas an increase in the number of bacteria in the semidormant (*S*), and persistent (*N*) states occurred only indirectly as a result of bacterial transfer. The transfer rates of all MTP parameters except *B*_max_ were fixed; *B*_max_ was estimated and represents the bacterial growth capacity of the system. Patients were assumed to have stationary-phase infections at the start of treatment, which corresponded to 150 days after infection in a model-based setting. An IIV component in *B*_max_ was added to account for a different baseline in the bacterial load between patients. Further, residual variability was accounted for by applying two additive components on a log scale. One of the residual error components was implemented on all replicates (ε), whereas the other residual error component accounted for replicates from the same sputum sample (ε_repl_).

Model-predicted individual PK profiles were utilized, to let adequate individual drug exposures drive an effect on different effect sites in the MTP model. Potential effect sites were defined as inhibition of the growth of multiplying bacteria or stimulation of the death of multiplying, semidormant, or persistent bacteria. Included drug effect models were on/off (a constant fixed drug effect with exposures above 0 mg/liter), linear, maximum effect (*E*_max_), and sigmoidal *E*_max_.

The exposure-response relationship in the clinical data was investigated in four sequential steps, as described by Svensson and Simonsson (14). The first step incorporated univariate drug effects on the different effect sites, utilizing the previously mentioned effect models. To not exclude effect sites that were apparent only in combination, the second step included combination of all effect sites with at least a linear effect model. The third step reevaluated the most significant exposure-response relationship at each of the effect sites, retesting all effect parameters. As the final step, a backward elimination of all exposure-response parameters was performed, to exclude nonsignificant effect sites at a 1% significance level (ΔOFV > 6.63 for removal of one parameter).

To evaluate the impact of the fixed system-related parameters on the final CLO drug effect model, a sensitivity analysis was conducted. Each parameter was empirically subject to a change of 15, 80, 120, and 185% of the original *in vitro* estimate, followed by reestimation of the drug effect and comparison of OFV (see Table S1 in the supplemental material).

### Statistical analysis.

The models were primarily selected on the basis of VPCs, the difference in the OFV, parameter precision, diagnostic plots, and scientific plausibility. Minimizing the OFV has been interpreted as maximizing the likelihood of the estimated model parameters, given the clinical data, using the first-order conditional estimation method with interaction. Model parameters and OFV were estimated using the software NONMEM (version 7.3; Icon Development Solutions, Hanover, MD) (43). A nested hierarchical model with the addition or exclusion of one parameter was considered to be statistically significant, at a 5% significance level, if OFV decreased by at least 3.84 for 1 degree of freedom (χ^2^ distribution). All diagnostic plots and data visualizations were performed using the R package Xpose (version 4.5.2; Department of Pharmaceutical Biosciences, Uppsala University, Uppsala, Sweden; http://xpose.sourceforge.net/). VPCs were generated using Pearl-Speaks-NONMEM (PsN; version 4.3.2; Department of Pharmaceutical Biosciences, Uppsala University; https://uupharmacometrics.github.io/PsN/), using 1,000 simulations (44). VPCs were assessed, in order to evaluate the 95% confidence intervals for the median and the 90th and 10th percentiles of the model-simulated data. As the drug effect evaluation was driven by individual PK profiles, each patient’s observed profile and the model-predicted PK profile were assessed using the same R package used for the diagnostic plots and data visualization. In addition, a 1,000-sample bootstrap using PsN was utilized to generate nonparametric 90% confidence intervals for all parameters in the final models (44) (https://uupharmacometrics.github.io/PsN/). Adequate tracking of the record and comparison of the models was maintained using Pirana software (version 2.9.7; Pirana Software & Consulting; http://www.pirana-software.com/) (45).

### Data availability.

The NONMEM code for the PK models and the PK-PD models can be found in the supplemental material. All relevant data are available from the authors upon reasonable request.

## Supplementary Material

Supplemental file 1
