# Supplementary material for: Drug Effect of Clofazimine on Persisters Explains an Unexpected Increase in Bacterial Load in Patients
Source: Antimicrob Agents Chemother. 2020 Apr 21;64(5):e01905-19. doi: 10.1128/AAC.01905-19 (PMC7179644; doi:10.1128/AAC.01905-19)
Supplement: Supplemental file 1 [file AAC.01905-19-s0001.pdf]

Supplementary information

Drug effect of clofazimine on persisters explain an unexpected increase in bacterial load from patients

Authors: Alan Faraj[a], Robin J. Svensson[a], Andreas H. Diacon[b,c,] Ulrika S.H. Simonsson[a*]

Affiliations:

[a] Department of Pharmaceutical Biosciences, Uppsala University, Uppsala, Sweden

[b] TASK Applied Science, Cape Town, South Africa

[c] Division of Physiology, Faculty of Medicine and Health Sciences, Stellenbosch University, Tygerberg, South Africa

***Correspondence:**

Ulrika SH Simonsson (ulrika.simonsson@farmbio.uu.se)

Overview of supplementary information

Supplementary Text File 1. Final NONMEM model code file for pyrazinamide pharmacokinetics

Supplementary Text File 2. Final NONMEM model code file for clofazimine pharmacokinetics

Supplementary Text File 3. Final NONMEM model code file for pyrazinamide pharmacokinetics-pharmacodynamics

Supplementary Text File 4. Final NONMEM model code file for clofazimine pharmacokinetics-pharmacodynamics

Supplementary Fig. S1. Individual pharmacokinetic profiles of all patients receiving (a) clofazimine and (b) pyrazinamide.
Supplementary Fig. S2. Typical bacterial simulations of the different sub-states of bacteria.

Supplementary Fig. S3. Observed pharmacokinetic data from day 14 showing each patients exposure.

Supplementary Table S1. Sensitivity analysis for the final model of clofazimine drug effect.

Supplementary Text 1:

Text File. Final NONMEM model file for pyrazinamide pharmacokinetics

```
$PROBLEM PZA pop PK
$INPUT ID TIME DV AMT EVID TAD RATE SEX WT
$DATA data.csv IGNORE=@
$SUBROUTINE ADVAN 13 TOL=9
$MODEL
COMP=(DEPOT, DEFDOSE)
COMP=(CENTRAL)
$MIX
NSPOP=2
P(1) = THETA(7)                    ; Proportion KA(1)
P(2) = 1 - THETA(7)                ; Proportion KA(2)
$PK
Q1 = 0
Q2 = 0
IF (MIXNUM.EQ.1) Q1=1
IF (MIXNUM.EQ.2) Q2=1
EST=MIXEST
TVCL = THETA(1) + THETA(10)*(WT - 48)
TVV = THETA(2) + THETA(9)*(WT-48) + THETA(11)*SEX            ; SEX=1 male, SEX=0 female
TVKA1 = THETA(3)
TVADDE = THETA(4)
TVPROPE = THETA(5)
TVD1 = THETA(6)
TVP1 = THETA(7)
TVKA2 = THETA(8)
TVWTV = THETA(9)
TVWTCL = THETA(10)
TVSV = THETA(11)
IOVCL = 0
```

```
IF(DAY.EQ.0) IOVCL=ETA(4)
IF(DAY.EQ.1) IOVCL=ETA(5)
IF(DAY.EQ.2) IOVCL=ETA(6)
IF(DAY.EQ.7) IOVCL=ETA(7)
IF(DAY.EQ.13) IOVCL=ETA(8)
IOVKA = 0
IF(DAY.EQ.0) IOVKA=ETA(9)
IF(DAY.EQ.1) IOVKA=ETA(10)
IF(DAY.EQ.2) IOVKA=ETA(11)
IF(DAY.EQ.7) IOVKA=ETA(12)
IF(DAY.EQ.13) IOVKA=ETA(13)
CL = TVCL*EXP(ETA(1)+IOVCL)
V  = TVV*EXP(ETA(2))
D1 = TVD1*EXP(ETA(3))
KA1 = TVKA1*EXP(IOVKA)
KA2 =  TVKA2*EXP(IOVKA)
KA = ((Q1 * KA1) + (Q2 * KA2))
KE=CL/V
S2=V
$DES
DADT(1) = -KA*A(1)
DADT(2) = KA*A(1)-KE*A(2)
$ERROR
; Residual error model specification
IPRED = A(2)/V
IRES = DV - IPRED
W=SQRT(THETA(4)**2 + THETA(5)**2*IPRED*IPRED)
IWRES = IRES/W
Y = IPRED+W*EPS(1)
IF(Y.LT.0) Y = 0.0001
$SIGMA 1 FIX
$THETA (0,3.42,34)                                    ; CL/F
```

```
$THETA (0,29,290)                          ; V/F
$THETA (0,3.56,9)                          ; KA1
$THETA (0,1.89,19)                         ; Add error
$THETA (0,0.0907,0.9)                      ; Prop error
$THETA (0,0.290,2.9)                       ; Dur of zero ord input into dose comp
$THETA (0,0.556,5.7)                       ; P1 proportion
$THETA (0,1.25,12.5)                       ; KA2
$THETA (0,0.433,4.3)                       ; WT on V/F
$THETA (0,0.0545,0.55)                     ; WT on CL/F
$THETA  (0,4.55,45.5)                      ; SEX on V/F
$OMEGA BLOCK(2)
0.0351                                     ; IIV CL/F
0.01 0.0251                                ; Covariance and IIV V/F
$OMEGA (0.957)                             ; IIV D1
$OMEGA BLOCK(1) 0.0238                     ; IOV CL/F
$OMEGA BLOCK(1) SAME
$OMEGA BLOCK(1) SAME
$OMEGA BLOCK(1) SAME
$OMEGA BLOCK(1) SAME
$OMEGA BLOCK(1) 0.623                      ; IOV KA
$OMEGA BLOCK(1) SAME
$OMEGA BLOCK(1) SAME
$OMEGA BLOCK(1) SAME
$OMEGA BLOCK(1) SAME
$SIGMA 1 FIX
$EST MAXEVAL = 0 POSTHOC
$TABLE ID TIME TAD DV IPRED IWRES  EVID     NOPRINT ONEHEADER
FILE=sdtab
$TABLE ID CL V KA KA1 KA2 D1 P1            NOPRINT ONEHEADER FILE=patab
$TABLE ID  WT                              NOPRINT ONEHEADER FILE=cotab
$TABLE ID SEX AMT                          NOPRINT ONEHEADER FILE=catab
```

Supplementary Text 2:

Text File. Final NONMEM model file for clofazimine pharmacokinetics

```
$PROBLEM CLO PopPK
$INPUT ID TIME DV AMT EVID TAD AGE SEX WT
$DATA data.csv IGNORE=@
$SUBROUTINE ADVAN 13 NTOL=9
$MODEL
COMP=(1)
COMP=(2)
COMP=(3)
$PK
TVCL = THETA(1)
TVKA = THETA(2)
TVV = THETA(3)
TVV2 = THETA(4)
TVQ = THETA(5)
TVALAG1 = THETA(6)
TVADDE = THETA(7)
TVPROPE = THETA(8)
TVF1 = 1
IOVF1 = 0
IF(DAY.EQ.0) IOVF1=ETA(4)
IF(DAY.EQ.1) IOVF1=ETA(5)
IF(DAY.EQ.2) IOVF1=ETA(6)
IF(DAY.EQ.7) IOVF1=ETA(7)
IF(DAY.EQ.13) IOVF1=ETA(8)
CL = TVCL*EXP(ETA(1))
KA = TVKA*EXP(ETA(2))
V = TVV*EXP(ETA(3))
V2 = TVV2
Q = TVQ
ALAG1 = TVALAG1
```

```
F1 = TVF1*EXP(IOVF1)
KE=CL/V
K12=Q/V
K21=Q/V2
S2=V
; INITIALIZATION OF COMPARTMENTS
A_0(1) = 0
A_0(2) = 0
A_0(3) = 0
$DES
DADT(1) = -KA*A(1)
DADT(2) = KA*A(1)-KE*A(2)-K12*A(2)+K21*A(3)
DADT(3) = K12*A(2)-K21*A(3)
$ERROR
; OUTPUT
ACOMP = A(1)
CCOMP = A(2)/V
PCOMP = A(3)/Q
IPRED = A(2)/V
IRES = DV - IPRED
W=SQRT(THETA(7)**2 + THETA(8)**2*IPRED*IPRED)
IWRES = IRES/W
Y = IPRED+W*EPS(1)
$THETA (0,13)            ; CL
$THETA (0,0.825)         ; KA
$THETA (0,1370)          ; V
$THETA (0,10500)         ; V2
$THETA (0,48)            ; Q
$THETA (0,0.7)           ; ALAG
$THETA (0,0 FIXED)       ; Additive error
$THETA (0,0.185)         ; Proportional error
;IIVs
```

```
$OMEGA (0.7)
$OMEGA (0.2)
$OMEGA (0.09)
;IOVS
$OMEGA BLOCK(1) 0.2
$OMEGA BLOCK(1) SAME
$OMEGA BLOCK(1) SAME
$OMEGA BLOCK(1) SAME
$OMEGA BLOCK(1) SAME
$SIGMA 1 FIX

$EST MAXEVAL = 9999 METHOD = 1 INTER
$COV PRINT = E MATRIX = S
$TABLE ID TIME TAD IPRED IWRES EVID CCOMP ACOMP PCOMP        NOPRINT
ONEHEADER FILE=sdtab
$TABLE ID CL V V2 Q KA  ALAG                                  NOPRINT
ONEHEADER FILE=patab
$TABLE ID AGE WT                                              NOPRINT
ONEHEADER FILE=cotab
$TABLE ID SEX                                                 NOPRINT
ONEHEADER FILE=catab
```

Supplementary Text 3:

Text File. Final NONMEM model file for pyrazinamide pharmacokinetics-pharmacodynamics

```
$PROBLEM Multistate Pharmacometric model applied to pyrazinamide EBA
$INPUT ID TIME NDV DV EVID AMT DAY DOSE L2 REP LOG10CFU IKA ICL ID1 IV
$DATA data.csv IGNORE=@
$SUBROUTINE ADVAN 13 TOL=9
$MODEL
COMP=(DEPOT, DEFDOSE)
COMP=(CENTRAL)
COMP=(FBAC)
COMP=(SBAC)
COMP=(NBAC)
$PK
CL = ICL
V   = IV
KA = IKA
D1 = ID1
; MTP system parameters
TVKG = THETA (1)                          ; Growth rate, F
TVKFSLIN = THETA(2)                        ; Time dependent, F to S
TVKFN = (THETA(3)/10000000)        ; F to N rate
TVKSF = THETA(4)                           ; S to F rate
TVKSN = THETA(5)                           ; S to N rate
TVKNS = THETA(6)                           ; N to S rate
TVBMAX = (THETA(7)*1000000000)              ; Carrying capacity
TVF0 = THETA(8)                                  ; Initial bacterial numb F state
TVS0 = THETA(9)                                  ; Initial bacterial numb S state
; Drug effect parameters
TVSDK = THETA(10)                                ; Killing of S state
KG = TVKG
KFSLIN = TVKFSLIN
```

```
KFN = TVKFN

KSF = TVKSF

KSN = TVKSN

KNS = TVKNS

BMAX = TVBMAX*EXP(ETA(1))

F0 = TVF0

S0 = TVS0

SDK = TVSDK

KE=CL/V

S2=V

; INITIALIZATION OF COMPARTMENTS

A_0(1) = 0

A_0(2) = 0

A_0(3) = 4.1

A_0(4) = 9770

A_0(5) = 0

$DES

GROWTH = KG*LOG(BMAX/(A(3)+A(4)+A(5)))          ; GOMPERTZ FUNCTION

IF(GROWTH.LT.0) GROWTH=0                         ; KEEP GROWTH FROM
TURNING NEGATIVE

KFS = (KFSLIN*T)/24                              ; KFSLIN IS SCALED TO DAYS

IF(DOSE.GT.0) THEN

PYRCP = A(2) / V                                 ; PYRAZINAMIDE PLASMA
CONCENTRATION

ELSE

PYRCP = 0

ENDIF

IF(PYRCP.GT.0) THEN

DSK = SDK*PYRCP

ELSE

DSK=0

ENDIF

DADT(1) = -KA*A(1)
```

```
DADT(2) = KA*A(1)-KE*A(2)
; MTP
DADT(3)=( A(3)*GROWTH+KSF*A(4)-KFS*A(3)-KFN*A(3) )/24                    ; F
DADT(4)=( KFS*A(3)+KNS*A(5)-KSN*A(4)-KSF*A(4)-DSK*A(4) )/24         ; S
DADT(5)=( KSN*A(4)+KFN*A(3)-KNS*A(5) )/24                                         ; N
$ERROR
; OUTPUT
ACOMP = A(1)
CCOMP = A(2)/V
FBAC    = A(3)
SBAC    = A(4)
NBAC   = A(5)
CFU      = A(3)+A(4)
IPRED   = LOG(A(3)+A(4))
IRES     = DV-IPRED
ADD1   = SQRT(SIGMA(1,1))
ADD2  = SQRT(SIGMA(2,2))
SD       = SQRT(ADD1*ADD1+ADD2*ADD2)
IWRES= IRES/SD
IF(REP==1) Y = IPRED+EPS(1)+EPS(2)                              ; Replicate 1
IF(REP==2) Y = IPRED+EPS(1)+EPS(3)                              ; Replicate 2
$THETA (0.206 FIXED)                                                      ; KG
$THETA (0.00166 FIXED)                                                   ; KFSLIN
$THETA (8.97 FIXED)                                                        ; KFN
$THETA (0.0145 FIXED)                                                     ; KSF
$THETA (0.186 FIXED)                                                       ; KSN
$THETA (0.00123 FIXED)                                                   ; KNS
$THETA (0,2.61)                                                              ; BMAX*10000000
$THETA (4.1 FIXED)                                                          ; F0
$THETA (9770 FIXED)                                                        ; S0
$THETA (0, 0.106)                                                           ; SDK
$OMEGA (0,2.3)                                                              ; IIV BMAX
```

; Residual error model specification

$SIGMA 1.20 ; ADD COMMON ERROR

$SIGMA BLOCK(1) 5.35E-02 ; ADD REPLICATE ERROR, for repl 1

$SIGMA BLOCK(1) SAME ; ADD REPLICATE ERROR, for repl 2

$EST MAXEVAL = 9999 METHOD = 1 INTER

$COV PRINT = E

$TABLE ID TIME DAY REP IPRED IWRES CFU LOG10CFU FBAC SBAC NBAC CCOMP ACOMP EVID   NOPRINT ONEHEADER FILE=sdtab

$TABLE ID CL V KA D1 SDK BMAX KG KFSLIN KFS KFN KSF KSN KNS NOPRINT ONEHEADER FILE=patab

$TABLE ID
NOPRINT ONEHEADER FILE=cotab

$TABLE ID
NOPRINT ONEHEADER FILE=catab

Supplementary Text 4:

Text File. Final NONMEM model file for clofazimine pharmacokinetics-pharmacodynamics

```
$PROBLEM Multistate Pharmacometric model applied to clofazimine

$INPUT ID TIME NDV DV EVID AMT DAY DOSE L2 REP LOG10CFU IETA1 IETA2
IETA3 IETA4 IETA5 IETA6 IETA7 IETA8

$DATA data.csv IGNORE=@

$SUBROUTINE ADVAN 13 TOL=9

$MODEL

COMP=(DEPOT, DEFDOSE)

COMP=(CENTRAL)

COMP=(PERIP)

COMP=(FBAC)

COMP=(SBAC)

COMP=(NBAC)

$PK

; Specify Typical PK parameters

TVCL     = 12.47

TVKA     = 0.67

TVV      = 1138

TVV2     = 8062

TVQ      = 63.32

TVALAG1 = 0.6241

TVF1     = 1

; MTP system parameters

TVKG = THETA (1)                   ; Growth rate, F

TVKFSLIN = THETA(2)                ; Time dependent, F to S

TVKFN = (THETA(3)/10000000)        ; F to N rate

TVKSF = THETA(4)                   ; S to F rate (Days-1)

TVKSN = THETA(5)                   ; S to N rate (Days-1)?

TVKNS = THETA(6)                   ; N to S rate (Days-1)?

TVBMAX = (THETA(7)*1000000000) ; Carrying capacity (CFU/ml)

TVF0 = THETA(8)                    ; Initial bacterial numb F state
```

```
TVS0 = THETA(9)                    ; Initial bacterial numb S state
 ; Drug effect parameters
TVNDK = THETA(10)                  ; Linear effect on N state

; IOV of PK driven from dataset.
IOVF1 = 0
IF(DAY.EQ.0) IOVF1=IETA4
IF(DAY.EQ.1) IOVF1=IETA5
IF(DAY.EQ.2) IOVF1=IETA6
IF(DAY.EQ.7) IOVF1=IETA7
IF(DAY.EQ.13) IOVF1=IETA8
CL      = TVCL*EXP(IETA1)
KA      = TVKA*EXP(IETA2)
V       = TVV*EXP(IETA3)
V2      = TVV2
Q       = TVQ
ALAG1 = TVALAG1
F1      = TVF1*EXP(IOVF1)
KE      =CL/V
K12     =Q/V
K21     =Q/V2
S2      =V
KG      = TVKG
KFSLIN = TVKFSLIN
KFN     = TVKFN
KSF     = TVKSF
KSN     = TVKSN
KNS     = TVKNS
BMAX  = TVBMAX*EXP(ETA(1))
F0      = TVF0
S0    = TVS0
NDK = TVNDK
```

```
; INITIALIZATION OF COMPARTMENTS
A_0(1) = 0
A_0(2) = 0
A_0(3) = 0
A_0(4) = 4.1
A_0(5) = 9770
A_0(6) = 0
$DES
GROWTH = KG*LOG(BMAX/(A(4)+A(5)+A(6)))    ; GOMPERTZ FUNCTION
IF(GROWTH.LT.0) GROWTH=0                         ; KEEP GROWTH FROM TURNING
NEGATIVE
KFS = (KFSLIN*T)/24                                     ; KFSLIN IS SCALED TO DAYS
IF(DOSE.GT.0) THEN
CLOCP = A(2) / V                                        ; CLOFAZIMINE PLASMA
CONCENTRATION (MG/L) in CCOMP
ELSE
CLOCP = 0
ENDIF

IF(CLOCP.GT.0) THEN
ND = NDK*CLOCP                                        ; INCREASED DEATH RATE, N,
SLOPE
ELSE
ND = 0
ENDIF
; PK
DADT(1) = -KA*A(1)
DADT(2) = KA*A(1)-KE*A(2)-K12*A(2)+K21*A(3)
DADT(3) = K12*A(2)-K21*A(3)
; MTP
DADT(4)=( A(4)*GROWTH+KSF*A(5)-KFS*A(4)-KFN*A(4) )/24        ; F state
DADT(5)=( KFS*A(4)+KNS*A(6)-KSN*A(5)-KSF*A(5) )/24             ; S state
DADT(6)=( KSN*A(5)+KFN*A(4)-KNS*A(6)-ND*A(6) )/24              ; N state
```

```
$ERROR
; OUTPUT
ACOMP = A(1)
CCOMP = A(2)/V
PCOMP = A(3)/Q
FBAC = A(4)
SBAC = A(5)
NBAC = A(6)
CFU = A(4)+A(5)
IPRED    = LOG(A(4)+A(5))
IRES     = DV-IPRED
ADD1           = SQRT(SIGMA(1,1))
ADD2           = SQRT(SIGMA(2,2))
SD       = SQRT(ADD1*ADD1+ADD2*ADD2)
IWRES    = IRES/SD
IF(REP==1) Y = IPRED+EPS(1)+EPS(2)                        ; Replicate 1
IF(REP==2) Y = IPRED+EPS(1)+EPS(3)                         ; Replicate 2

$THETA (0.206 FIXED)                                        ; KG
$THETA (0.00166 FIXED)                                      ; KFSLIN
$THETA (8.97 FIXED)                                          ; KFN
$THETA (0.0145 FIXED)                                       ; KSF
$THETA (0.186 FIXED)                                         ; KSN
$THETA (0.00123 FIXED)                                      ; KNS
$THETA (0,0.055)                                           ; BMAX*10000000
$THETA (4.1 FIXED)                                          ; F0
$THETA (9770 FIXED)                                        ; S0
$THETA (0,1.5)                                            ; NDK
$OMEGA 1.73                                             ; IIV BMAX
; Residual error model specification
$SIGMA  1.20E+00                                        ; ADD COMMON ERROR
```

$SIGMA BLOCK(1) 5.35E-02   ; ADD REPLICATE ERROR, for repl 1

$SIGMA BLOCK(1) SAME   ; ADD REPLICATE ERROR, for repl 2

$EST MAXEVAL = 9999 METHOD = 1 INTER

$COV PRINT = E

$TABLE ID TIME DAY IPRED PRED IWRES CFU LOG10CFU FBAC SBAC NBAC CCOMP ACOMP PCOMP EVID NOPRINT ONEHEADER FILE=sdtab

$TABLE ID CL V V2 KA BMAX KG KFS KFSLIN KFN KSF KSN KNS NDK NOPRINT ONEHEADER FILE=patab

$TABLE ID
NOPRINT ONEHEADER FILE=cotab

$TABLE ID
NOPRINT ONEHEADER FILE=catab

Supplementary Figures

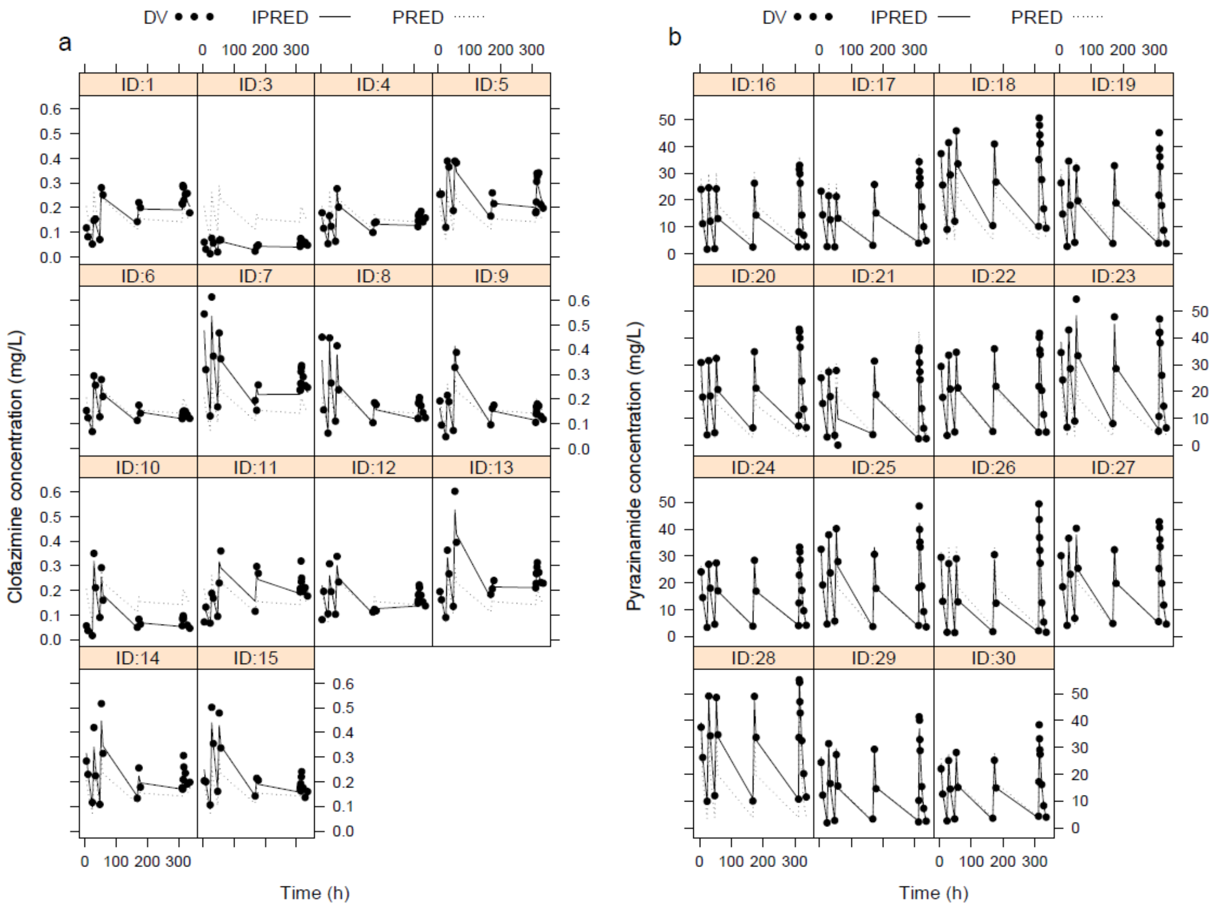

Figure S1. Individual pharmacokinetic profiles of all patients receiving (a) clofazimine and (b) pyrazinamide. Black circles, solid line and dotted line represents observed concentrations (DV), individual model-predicted concentrations (IPRED) and population model-predicted concentrations (PRED), respectively.

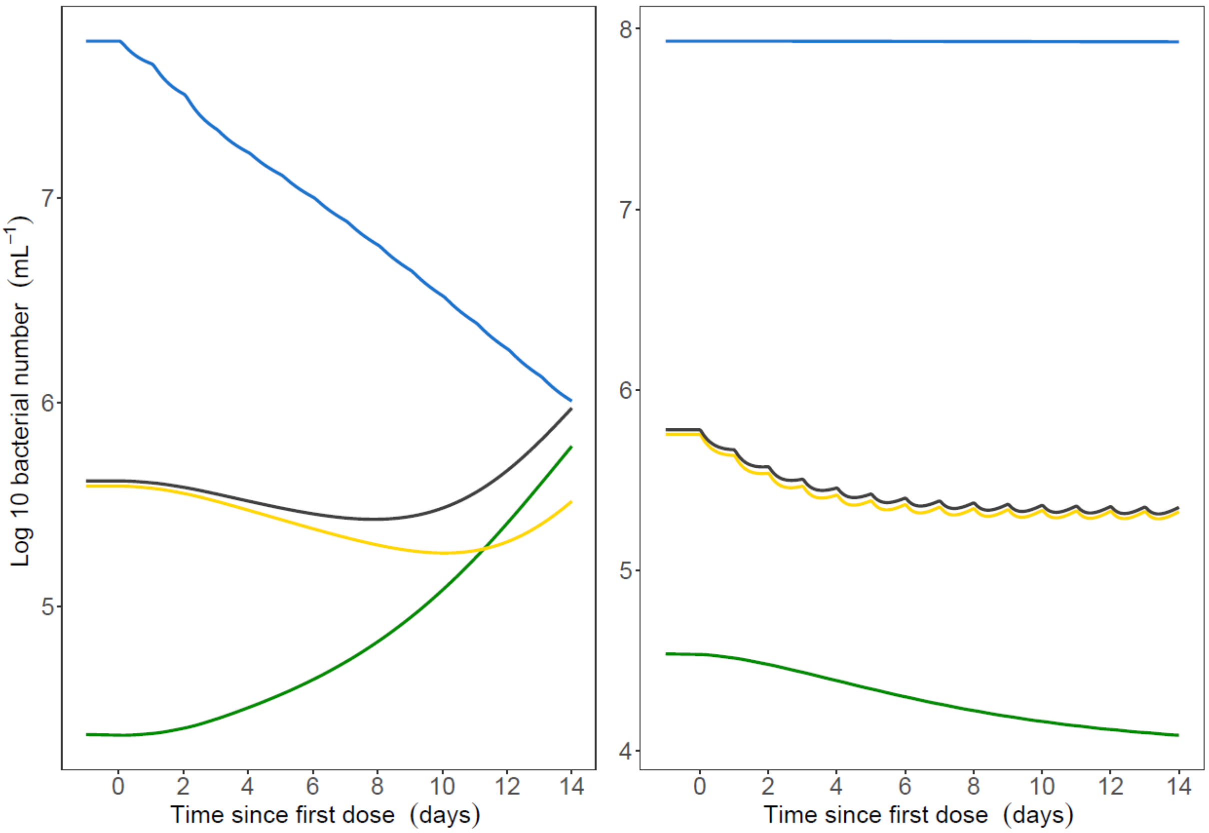

Figure S2. Log10 typical bacterial simulations from the final models over 14 days. The blue, yellow and green lines represents non-, slow- and fast-multiplying bacterial numbers, respectively. The grey line represent CFU counts consisting of fast- and slow-multiplying bacteria.

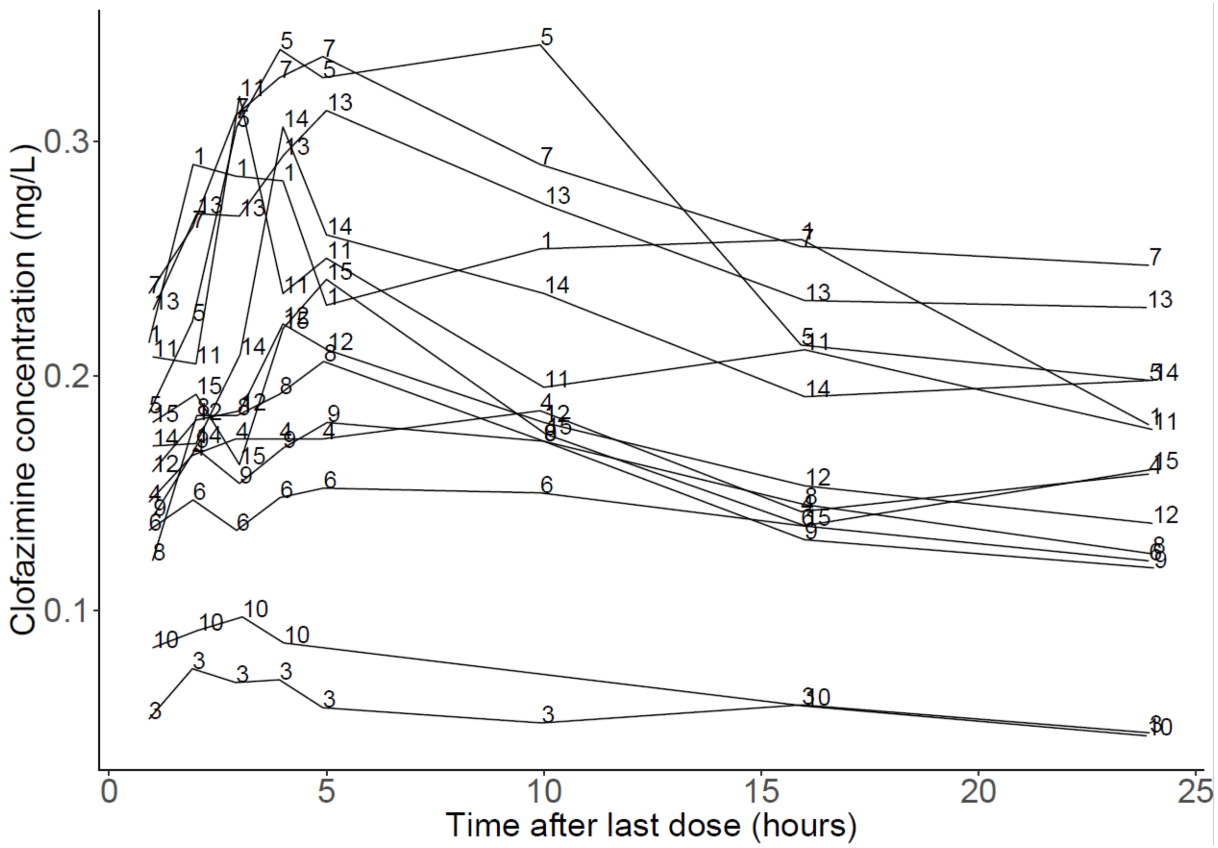

Figure S3. Observed clofazimine pharmacokinetic data from day 14 showing each patients exposure. Numbers represents observation point and patient ID.

Supplementary Table 1:

Table S1. Sensitivity analysis of the final model for clofazimine drug effect.

Parameter	ΔOFV as compared to the final model	Fixed Value	% of original value
k_{SN}	-2.15	2.79×10^{-2}	15
	-1.72	1.49×10^{-1}	80
	1.83	2.23×10^{-1}	120
	7.57	3.44×10^{-2}	185
k_{NS}	0.44	1.85×10^{-4}	15
	0.07	9.84×10^{-4}	80
	-0.06	1.48×10^{-3}	120
	-0.21	2.28×10^{-3}	185
k_{FN}	0	1.34×10^{0}	15
	0	7.18×10^{0}	80
	0	10.8×10^{0}	120
	0	16.6×10^{0}	185
k_{SF}	11.49	2.18×10^{-3}	15
	1.19	1.16×10^{-2}	80
	-0.90	1.74×10^{-2}	120
	-2.69	2.68×10^{-2}	185
k_{FSLIN}	-2.56	2.49×10^{-4}	15
	-1.40	1.33×10^{-3}	80
	1.31	1.99×10^{-3}	120
	4.51	3.07×10^{-3}	185
k_G	9.43	3.09×10^{-2}	15
	1.19	1.65×10^{-1}	80
	-1.05	2.47×10^{-1}	120
	-3.17	3.81×10^{-1}	185

All system related parameters was subject to change in this sensitivity analysis. % of original value refers to the estimates reported in Table 1.
